# From trade-off to synergy: how nutrient status modulates plant resistance to herbivorous insects?

**DOI:** 10.1007/s44307-024-00045-5

**Published:** 2024-10-08

**Authors:** Zhi-Xing Li, Jin-Fang Tan, Nan Yao, Ruo-Han Xie

**Affiliations:** 1 State Key Laboratory of Biocontrol, Guangdong Provincial Key Laboratory of Plant Stress Biology, School of Agriculture and Biotechnology, Shenzhen Campus of Sun Yat-Sen University, Shenzhen, 518107 China; 2 State Key Laboratory of Biocontrol, Guangdong Provincial Key Laboratory of Plant Stress Biology, School of Life Sciences, Sun Yat-Sen University, Guangzhou, 510275 China

**Keywords:** Plant–insect interaction, Mineral nutrients, Growth-defense trade-off, Volatile organic compounds, Integrated pest management

## Abstract

The principle of the “growth-defense trade-off” governs how plants adjust their growth and defensive strategies in response to external factors, impacting interactions among plants, herbivorous insects, and their natural enemies. Mineral nutrients are crucial in modulating plant growth and development through their bottom-up effects. Emerging evidence has revealed complex regulatory networks that link mineral nutrients to plant defense responses, influencing the delicate balance between growth and defense against herbivores. This review aims to summarize recent advances that elucidate the impact of nutrient availability on plant defense responses. Particularly, we focus on how nutrient status shapes plant resistance to herbivores, delving into the molecular mechanisms underlying this physiological process. Moreover, the interplay between mineral nutrients and various herbivore defense mechanisms, including physical protection, plant hormone signaling, defensive metabolite production, and volatile organic compound emissions that deter herbivores or attract their natural enemies, are discussed. This comprehensive review sets the stage for future investigations into the intricate crosstalk between nutrient signaling and plant defense responses, which serves as a central mechanism to guide sustainable pest management approaches, thereby promoting balanced agroecosystem health and enhancing plant ecosystem productivity and resilience.

## Introduction

Plants are crucial primary producers in ecosystems, but navigate a complex landscape of challenges that include intense competition and various abiotic and biotic stresses (Nawaz et al. 2023). Insects represent one of the most significant threats to plant survival due to their abundance and diversity (Erb and Reymond 2019). Throughout the protracted co-evolutionary arms race between plants and insects, plants have evolved intricate co-adaptation mechanisms and strategies to cope with the pressures imposed by herbivorous insects (Solé 2020; Cui et al. 2022).

The dynamic interplay between plant growth and defense mechanisms reflects the delicate balance of resource allocation between growth and defense responses, a topic of enduring interest among researchers (He et al. 2022). Among their sophisticated regulatory systems, mineral nutrition—a limited resource for both insects and host plants—exerts bottom-up effects on plant-herbivore-natural enemy multitrophic interactions within ecosystems (Han et al. 2022). Specifically, plant mineral nutrient traits can influence defenses in several ways, such as by strengthening structural defenses that serve as physical barriers against insect feeding and oviposition (Lewandowska et al. 2020; Liu et al. 2024), regulating the production of defensive metabolites that establish antifeedant, repellent, and toxic effects against herbivorous insects (Ali et al. 2019), and triggering the emission of volatile organic compounds (VOCs) that signal and attract natural herbivore enemies (Dudareva et al. 2013; Erb et al. 2021). However, the present knowledge regarding the effects of nutrient availability on plant resistance to insects is largely based on field observations and practical experience, which often yield conflicting results. As indicated in a meta-analysis, plants suppress herbivore populations not only through average quality but also via heterogeneous nutrient levels (Wetzel et al. 2016), suggesting that there is intricate crosstalk between nutrient signaling pathways and defense responses elicited upon herbivore attack in plants. Recent advances are yet to be comprehensively synthesized to illustrate how nutrient status governs the immune system of the plant under herbivorous stress.

To address this gap, this review summarizes current knowledge on the intricate relationship between plant nutrient status and defense responses in the context of interactions with herbivorous insects. It explores the multifaceted nature of mineral nutrition and its effects on plant physical, chemical, and inducible defenses against herbivorous insects. The focus of this review is on the mechanisms underlying the crosstalk between nutrient signaling and various defense responses. Ultimately, we offer our viewpoints on the prospective developments on the future of nutrient-mediated plant insect resistance, emphasizing the importance of considering fertilization strategies for optimizing integrated pest management in future agronomic practices.

## Trade-off strategy for nutrition between plant growth and insect resistance

The strategic distribution of resources by plants for defense, growth, and reproduction, coupled with the differential expression of defensive traits in various tissues, is a central concept in plant defense theories (Meldau et al. 2012; Valim et al. 2020). Plants respond to herbivorous insect attacks by activating diverse defense mechanisms that alter the composition and use of internal mineral nutrients. This phenomenon often results in a “growth-defense trade-off” strategy. This is attributed to the fact that both growth and defense mechanisms require substantial resources. In response to herbivore attacks, plants reassign resource distribution from growth to defense. The growth-defense trade-off has likely evolved to avoid the inefficient use of resources for tissues likely to be consumed by pests (He et al. 2022). Thus, the growth-defense relationship is complex, and antagonistic effects may exist.

Taking nitrogen (N) as an example, due to the potential limitation of N as a nutrient, N pools are susceptible to fluctuating demands and intense intraspecific competition. Valim et al. (2020) demonstrated that plants modulate their defensive mechanisms by prioritizing the production of N-rich defense molecules and reallocating nitrogen resources between growth and reproduction under herbivore-induced stress. Numerous studies have suggested that leaf N levels correlate with herbivorous insect performance in plant–insect interactions (Joern et al. 2012). Nitrogen is seen as a measure of plant quality and a critical factor that can restrict the performance of herbivores (Comadira et al. 2015; Hansen et al. 2020). Plants with sufficient N levels exhibit enhanced vegetative growth, an expanded leaf area, and efficient photosynthetic rates; consequently, they are generally more attractive to insects (Prudic et al. 2005), Consistent findings were observed for phosphorus (P) in the larvae of Fall armyworm (FAW) (*Spodoptera frugiperda*), which preferentially selected host plants with high P:C ratios (Waldbauer et al. 1984).

Plants with high nutrient status also have a reduced ability to repel a broad spectrum of herbivores, including FAW (Wang et al. 2022b), *Spodoptera exigua* (Ren et al. 2013), and *Spodoptera litura* (Li et al.^2024^). This may be due to changes in their chemical profiles, such as a reduction in the content of insect-resistant substances. For instance, the application of N reduced the silicon (Si) content and relative water content (RWC) in rice plants, resulting in increased feeding by the brown planthopper (BPH) (Rashid et al. 2016). In contrast, lower N contents may decrease the insect’s preference and feeding capacity, slowing their growth and development. For instance, Li et al. (2024) demonstrated that limited N availability endowed tomato plants with resistance against *S. litura*, which could be linked to the biosynthesis and release of the volatile organic compound α‐humulene acting as a repellent. This phenomenon supports the adaptive growth hypothesis (AGH), which proposes that plants with higher C:N ratios prioritize survival, whereas plants with lower C:N ratios in nitrogen-rich environments prioritize growth (Zhang et al. 2020). Increased N input may selectively enhance plant growth and development, resulting in increased allocation of resources to primary metabolite synthesis pathways and increased protein and starch production. This may decrease the formation of specific defensive compounds in plants, creating a favorable environment for insects.

Nevertheless, some researchers have reached the opposite conclusion. *Oedaleus asiaticus*, a major locust of North Asia, was found to decrease in size and viability due to host plant N enrichment and protein-rich artificial meals (Cease et al. 2012). This result may be partially attributed to the different responses of herbivorous species to host plant nutrient levels (Cease et al. 2012; Le Gall et al. 2020; Virla et al. 2023).

## Contrasting roles of mineral nutrition on plant physical defenses against herbivorous insects

Plant physical resistance traits refer to morphological and structural adaptations, such as cell wall lignification, thick cuticles, waxes, and trichomes, which create a physical barrier that influences the feeding behavior and performance of insects (Liu et al. 2010; Gong and Zhang 2014). The cuticle of plant leaves acts as the first physical layer that protects aboveground plant tissues from attack and predation by herbivores, due to its impermeability and tough physical properties, which create an obstacle for insect herbivore attachment, feeding, and oviposition (Lewandowska et al. 2020; Liu et al. 2024). Investigations have highlighted the inverse relationship between N levels and the rigidity of plant epidermis (Sun et al. 2018). Typically, a higher N input promotes plant growth, but at the price of reduced lignin and waxy cuticle formation (Sun et al. 2020). Specifically, when plants were subjected to excessive N levels, delayed lignin deposition in the xylem cell wall reduced the thickness of the secondary cell wall, and decreased levels of key biopolymer components (cellulose and lignin) were observed (Jauset et al. 2000; Camargo et al. 2014; Zhang et al. 2017). Reducing N fertilizer application rates can significantly enhance plant resistance to lodging, a trait linked to increased stem lignification and the development of secondary cell walls. Therefore, this could partially explain the phenomenon whereby nitrogen-rich plants are susceptible to various insect attacks because the high N levels have weakened their structural barriers and they have become more vulnerable to invasion by herbivorous insects.

Potassium (K) nutrition plays the opposite role in the physical resistance of plants against insects. Adequate K supply tends to strengthen plant structures and improve characteristics such as stronger cuticles, reinforced cell walls, improved development of sclerenchyma tissues, and the stimulation of lignification and silicification. This strengthening of plant structures contributes significantly to the plant’s capacity to withstand damage from insect-feeding (Singh and Sood 2017). High K availability was associated with a decrease in survival rate, body weight, and pest populations, such as the striped stem borers *Chilo suppressalis* (SSB) and rice leaf folders *Cnaphalocrocis medinalis* (LF) (Holzmueller et al*.* 2007). Thus, K application is useful in eliminating damage to plants caused by stem borer larvae infestation in rice and has demonstrated effectiveness in boosting yields (Sarwar 2012).

Silicon is another nutrient that plays a vital role in enhancing plant physical resistance against insect pests, and this is particularly true for piercing-sucking insects (Mandlik et al. 2020). Silicon application leads to the development of hard and abrasive surfaces on wheat leaves, resulting in reduced crop damage from *Schizaphis graminum* and decreased insect performance (Goussain et al*.* 2005). Adult BPH exhibited significantly lower feeding selectivity and honeydew excretion on Si-treated rice compared with untreated rice (Yang et al. 2017). Moreover, silicification of plant tissues increases their hardness and abrasiveness, thereby reducing chewability and digestibility for chewing herbivores. This is corroborated by evidence showing that Si reduces the growth rate and food conversion efficiency of African armyworms (Massey and Hartley 2009).

## Crosstalk between nutrient signaling and defense responses triggered by herbivore attack

Despite the sophisticated physical defense responses of plants, insects can damage their barriers and introduce substances that impair the plant's defense and immune mechanisms (Acevedo et al. 2015; Duran-Flores & Heil 2016). Consequently, plants have evolved various other insect resistance mechanisms. A rapid and effective defense reaction hinges on accurately detecting herbivore assaults, converting these detections into signals that reconfigure cellular activities for defense. Among these plant–insect interactions, mineral nutrition plays an important role, possibly by modulating the crosstalk between early nutrient signaling and the triggering of a defense response.

### Jasmonic acid (JA) signaling

**Fig. 1:**
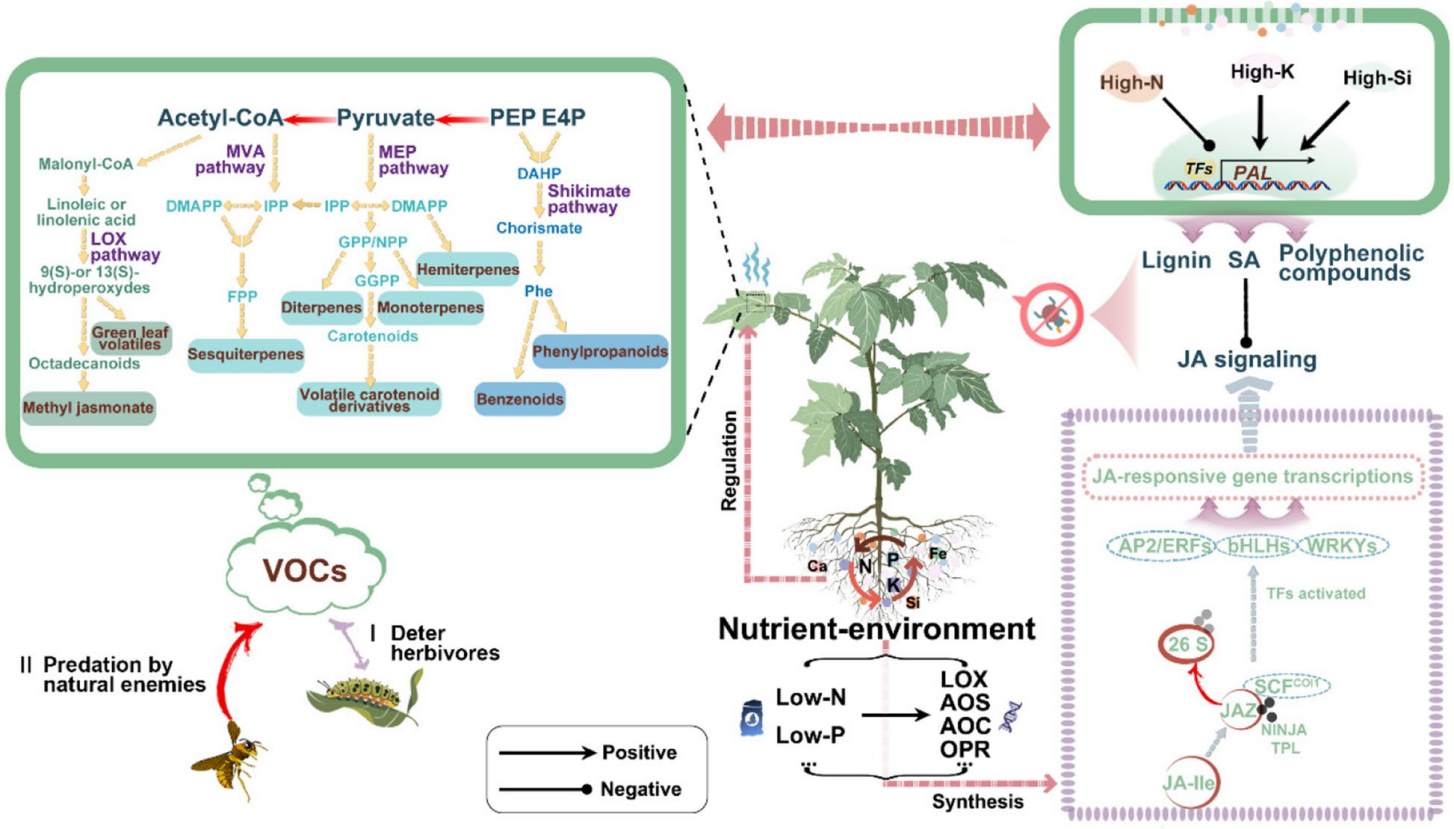
Nutrient cues modulate VOC formation and crosstalk with hormone signaling. On the left are the VOC biosynthetic pathways. The pathways involved include the shikimate/phenylalanine, mevalonic acid (MVA), methylerythritol phosphate (MEP), and the lipoxygenase (LOX) pathways. These pathways involve multiple enzymatic reactions, as depicted by stacked arrows. VOCs encompass a diverse array of compounds such as including benzenoids/phenylpropanoids, sesquiterpenes, monoterpenes, hemiterpenes, diterpenes, volatile carotenoid derivatives, and methyl jasmonate/green leaf volatiles, all set against a purple cloud backdrop. The effects of VOCs on herbivorous insects, I, deter herbivores; II, predation by natural enemies. Key acronyms include PEP (phosphoenolpyruvate), E4P (erythrose 4-phosphate), DAHP (3-deoxy-D-arabinoheptulosonate-7 phosphate), DMAPP (dimethylallyl pyrophosphate), IPP (isopentenyl pyrophosphate), GPP (geranyl pyrophosphate), NPP (neryl pyrophosphate), GGPP (geranylgeranyl pyrophosphate), and FPP (farnesyl pyrophosphate). On the right is a schematic illustration of the synthetic mechanisms of the JA signaling and phenylpropanoid pathways affected by nutrient availability. Abbreviations: High-N, high nitrogen; High-P, high phosphorus; High-K, high potassium; High-Si, high silicon; Low-N, low nitrogen; Low-P, low phosphorus; JA-Ile, jasmonic acid isoleucine; JAZ, jasmonate-zim; SCF, SKP1–CUL1–F-box protein E3 ubiquitin ligase

JA, a lipid-derived phytohormone, acts as a principal modulator of plant defenses against insect pests; its signaling networks link perception and early signaling with plant immune responses (Thines et al. 2007; Sheard et al. 2010; Erb and Reymond 2019). The induction of defense typically involves the accumulation of JA, along with the activation of a series of downstream signaling pathways (Malook et al*.* 2021; Monson et al. 2022). Signals derived from different nutrients can regulate the JA-dependent defense response by modulating its synthesis, metabolism, transport, and signal transduction. Among them, the JA signaling pathway is considered a pivotal route in modulating plant defenses against herbivorous insects (Nguyen et al. 2022). JA-Ile, the bioactive form of jasmonic acid in vascular plants, is recognized by a receptor complex that includes the F-box protein CORONATINE INSENSITIVE1 (COI1) and proteins from the JASMONATE ZIM DOMAIN (JAZ) family (Sheard et al. 2010; Chini et al. 2023; Hu et al. 2023). The ubiquitination and subsequent proteasomal degradation of JAZ proteins by the SCF^COI1^ complex liberate the basic helix-loop-helix (bHLH) transcription factor MYC2, initiating the activation of diverse JA-responsive genes (Schweizer et al. 2013; Wu et al. 2024) (Fig. 1).

Nitrogen deficiencies can confer rice resistance to SSB by increasing the levels of JA. In situations with low N availability, the accumulation of lignin in rice plants may impede the initiation of the JA signal during defense against herbivore infestation (Zheng et al. 2021). Insufficient N can also potentially enhance the response to herbivory by priming or increasing the induction of JA-related genes (*LOX6*, *AOS3*, *AOC*, and *OPR3*) and defense enzymes, resulting in heightened JA levels post-insect attack (Zheng et al. 2024). These results suggest that N deprivation can impact plant defense mechanisms through the activation of JA-mediated anti-herbivore responses.

The implications and outcomes of activating the JA pathway in reaction to P scarcity have also been clarified (Kong et al. 2021; Pandey et al. 2021). Phosphorus deficiency-induced heightened resistance to *Spodoptera littoralis*, indicating that the association between P deficiency and improved resistance to herbivory is consistent across various plant species. In both wild-type (WT) plants and phosphorus-deficient mutant *pho1 Arabidopsis*, P-deficiency induced an increase in the biosynthesis of JA and the bioactive conjugate JA-isoleucine, along with the JA signaling pathway, by enhancing the expression of *JAZ10*, *VSP2*, and *LOX2* (markers of the JA pathway). In particular, the *pho1* mutant demonstrates enhanced resistance to the generalist herbivore *S. littoralis* compared with the WT. Of note, the JA-mediated defensive response against herbivorous insects is altered in *Arabidopsis* mutants including the genes implicated in the production of inositol polyphosphate. The timing and kinetics of the changes in phosphoinositides and InsP3 induced by insect wounding align with the increased JA levels. In addition, *Arabidopsis ipk1-1* mutants, characterized by elevated InsP5 levels, exhibited enhanced defensive capabilities through COI1-driven processes, such as upregulated gene expression in response to injury, defense against caterpillars, and root growth inhibition by jasmonate (Mosblech et al. 2008, 2011). The role of VIH2 in the modulation of jasmonate signaling and the plant's defense against herbivorous insects has been delineated. VIH2-mediated inositol pyrophosphates function as essential cofactors of the COI1-JAZ receptor complex, which significantly contributes to jasmonate signaling and the modulation of jasmonate-induced defense mechanisms (Laha et al. 2015). Consequently, P deficiency may impact JA signaling pathways by regulating the inositol polyphosphate pool.

Though high N or P levels are known to compromise plant defense mechanisms against herbivorous insects, some nutrients may exert synergistic effects in enhancing insect resistance to insects. For instance, Si has been associated with heightened production of plant's chemical defenses against herbivorous insects, modulating the biosynthesis of such protective compounds, particularly through the JA-dependent defense response. Recent reviews have highlighted that Si enhances the activity of the JA signaling pathway in plants when they are subjected to herbivore damage (Reynolds et al. 2016; Debona et al. 2017; Alhousari and Greger 2018). Hall et al. (2019) revealed that Si acts as a physical trigger within the plant, leading to a brief and modest elevation in JA levels. Si pretreatment increases the accumulation of JA in rice, enhances the expression levels of defense marker genes, and elevates the activities of peroxidase, polyphenol oxidase, and trypsin protease inhibitors, thereby enhancing the resistance of rice to LF. Moreover, the silencing of key genes allene oxide synthase (*OsAOS*) and CORONATINE INSENSITIVE1 (*OsCOI1*) in the JA signaling pathway significantly reduces the deposition of Si and the expansion of Si cells in rice (Ye et al. 2013). Similarly, research on tomato-*Ralstonia solanacearum* interactions showed that Si application significantly enhanced JA synthesis under disease stress (Jiang et al. 2019). Moreover, Si application has been shown to upregulate the expression of Si channels *OsLsi1*, *OsLsi2*, and *OsLsi6*, which are also induced by both insect infestation and MeJA treatment (Singh et al. 2020). Collectively, these findings suggest a synergy relationship between nutrient status and plant resistance to herbivorous insects.

### Phenylpropanoid pathway

Lignin, salicylic acid (SA), and other polyphenolic compounds that play crucial roles in plant defense mechanisms against a wide range of pathogens and insect pests are all products of the phenylpropanoid pathway (He et al. 2020). The induction of lignin biosynthesis-related genes, such as phenylalanine ammonia-lyase (PAL) that encodes the initially committed enzyme in the phenylpropanoid pathway, was predominantly observed following the invasion of sap-sucking herbivores (Paulillo et al. 2000; Chaman et al. 2003; He et al. 2020) (Fig. 1). Plants subjected to high levels of N exhibited significantly lower expression of *PAL* following herbivore attack, indicating that N-rich plants may contain lower levels of lignin (Wang et al. 2022b). The opposite conclusion was reached in maize plants, where increased N fertilization increased the total phenolic content by inducing *PAL* activity, ultimately decreasing the fitness of *Ostrinia furnacalis* on the host plant (Xu et al. 2019a). The application of K also significantly boosted the levels of endogenous SA and the activity of *PAL* in potato leaves, thereby enhancing the plant's resistance to aphid infestations (Xu et al. 2019b). External supplementation of calcium (Ca) on kidney bean plants resulted in a notably enhanced resistance against *Frankliniella occidentalis* (Zeng et al. 2020). Silicon has also been shown to elevate SA levels in rice plants infested with BPH, concomitantly elevating the transcription of pivotal genes in SA synthesis, such as *OsPAL4*. In the Si uptake mutant *lsi1*, the enhancement of BPH resistance and the promotion of SA synthesis and accumulation by Si are significantly diminished or entirely negated (Lin et al. 2022a, b).

### Protease inhibitor

Plant protease inhibitors can suppress the activity of insect herbivores’ digestive proteases, thereby impeding the absorption of essential amino acids that are crucial for herbivore growth and development (Zhu-Salzman and Zeng 2015). For instance, soybean proteinase inhibitors impacted the activities of trypsin and chymotrypsin, and this hindered the growth of FAW larvae (Paulillo et al. 2000). The maize proteinase inhibitor can suppress the activity of two classes of insect digestive enzymes, including elastases and chymotrypsin (Quilis et al. 2014). The expression of *MPI*, which encodes the maize proteinase inhibitor protein, was notably enhanced in high-nitrogen maize under regular conditions. Conversely, upon herbivore attack, a significant increase in *MPI* expression was observed in low-nitrogen maize (Wang et al. 2022b). The effect of N application on herbivore-induced defenses in *Nicotiana attenuate* showed that a reduced N supply diminished the synthesis of N-dependent direct defenses, specifically trypsin proteinase inhibitors (TrypPI). Additionally, elevated transcript levels of TrypPI were detected in plants treated with silicon compared to those that were not treated (Singh et al. 2020).

### Defensive metabolites biosynthesis and accumulation

Recognition of herbivore damage and the subsequent triggering of signaling cascades leads to the production and buildup of defensive metabolites with insecticidal, repellent, and anti-digestive effects (Divekar et al. 2022).

Nitrogen has been acknowledged as an important signal that controls the generation of insect-defensive secondary metabolites (Wang et al. 2022b). For instance, N-rich compounds, such as alkaloids, have varied chemical structures and bioactivities, including antifeedant, repellent, and toxic effects on herbivorous insects (Ali et al. 2019). Plants with elevated levels of alkaloids show increased resistance to herbivory, as these compounds deter feeding or disrupt insect growth and development. On the other hand, phenolics, a well-established class of carbon-based secondary metabolites that can disrupt digestion, impede growth, and inhibit enzyme activity and cell division, also play key roles in N-mediated plant pest resistance processes. High levels of phenolics were detected in plants with insufficient N, and they depressed plant resistance (Ren et al. 2013). *Populus tremuloides* Michx was subjected to low-N fertilization and exhibited significantly higher levels of phenolics compared with plants with low fertilization, leading to reduced growth of *Choristoneura conflictana* (Walker), and aspen tortrix (Bryant et al. 1987). Similar findings were observed in rice when the N deficiency improved rice resistance to SSB via the accumulation of phenolic acids and flavonoids as secondary metabolites (Zheng et al. 2021).

Compared with N, P was reported to play a contrary role in mediating plant resistance to pests through the synthesis of phenolic metabolites. In potato plants, P depletion makes them less suitable for various insect pests as they have altered the production of phenolics (Abd el-aziz et al. 2008). The deficiency of essential nutrients required for the de novo synthesis of induced defensive compounds could account for this reduction (Gouinguené and Turlings 2002).

In recent years, the intimate connection between nutrient stress responses and the plant immune system has been documented in plant reactions to iron (Fe), which indicate a broad and intricate interplay between nutrient uptake and plant immune defenses (Sun et al. 2023; Cao et al. 2024). Hu et al. (2021) demonstrated that the inhibitory impact of benzoxazinoids, shikimic acid-derived secondary metabolites abundantly produced by grasses on FAW growth, contributed to plant resistance, was influenced by soil Fe status, and could be reversed. Benzoxazinoids inhibit herbivore growth under Fe-deficient conditions and when chelated Fe is present and promote herbivore growth when there is free Fe in the growth medium.

## Diverse influences of nutrition on plant-herbivore-natural enemy tri-trophic interactions mediated by VOCs

Accumulating defensive secondary metabolites serves as a potent defense mechanism and competitive tactic for plants to ward off herbivores (Jhu and Sinha 2022). Volatile organic compounds are synthesized through secondary metabolic processes and function as airborne chemical signals mediating intra- and interspecific interactions, especially in insect-plant interactions (Pichersky et al. 2006; Allmann and Baldwin 2010; Munawar et al. 2022). The synthesis pathways of plant VOCs are generally divided into three categories (Dudareva et al. 2013; Liu et al. 2023) (Fig. 1). VOCs derive their precursors from primary metabolism and are broadly categorized into four groups based on their biosynthetic pathways and chemical structures (Jin et al. 2023). These groups encompass terpenoids, phenylpropanoids/benzenoids, fatty acid derivatives, amino acid derivatives, and species- or genus-specific compounds that do not conform to these primary classifications (Dudareva et al. 2013).

In nature, tri-trophic interactions involving plants, herbivores, and natural enemies form fundamental components of ecosystems worldwide (Qin et al. 2023), in which plants are always the dominant providers of food or shelter to herbivores and cues to the natural enemy (Liu et al. 2024). This relationship could be mediated by volatile-based communications with plant VOCs serving as either direct defense molecules to deter insects, as discussed above, or indirect defense signals to recruit natural enemies of invading insects. The ecological regulatory effects of some identified important plant volatiles, including constitutive plant volatiles (CPV) and herbivore-induced plant volatiles (HIPVs), on herbivorous or natural enemy insects are summarized in Table 1.

**Table 1:** Ecological control of VOCs in tri-trophic interactions

Chemical name	Source		Species and regulation effect		References
	**CPV**	**HIPV**	**Herbivores**	**Natural enemies**	
(*Z*)-3-hexenyl butyrate, (*Z*)-3-hexenyl Acetate		*Nicotiana*	*Heliothis* *virescens*●		(De Moraes et al. 2001)
(*Z*)-3-hexenyl acetate		*Z. mays*	*S.exigua*●		(Engelberth et al. 2004)
Nicotine		*Nicotiana*	*F.occidentalis*●		(Delphia et al. 2007)
A mixture of (*Z*)-3-hexen-1-ol, 1-hexanol, benzaldehyde, (*E*)-*β*-farnesene and methyl salicylate, et al	*Vicia faba*		*Aphis fabae*◆		(Webster et al. 2008)
Mixture of (*Z*)-3-hexenal, (*E*)-2-hexenal, (*E*)-3-hexen-1-ol and (*Z*)-3-hexenyl acetate with a small amount of linalool and β-myrcene		*Z. mays*		*Trichogramma pretiosum*◆	(Peñaflor et al. 2011)
(*E*)-*β*-caryophyllene:DMNT: (*E*)-*β*-farnesene (100:78:9)	*Vitis vinifera*		*Lobesia botrana*◆		(Bruce and Pickett 2011)
Linalool	rice		*Nilaparvata lugens*●	*Anagrus nilaparvatae*◆	(Xiao et al. 2012)
2, 3‐butanediol	*Zea mays*		*S.littoralis*●	*Cotesia marginiventri*◆	(D’alessandro et al. 2014)
DMNT		*Gossypium* *hirsutum*	*S. littoralis*●		(Hatano et al. 2015)
*β*‐ocimene and *α*‐phellandrene		Broad Bean	*Acyrthosiphon pisum*	*Aphidius ervi*◆	(Takemoto and Takabayashi 2015)
1-Octen-3-ol	Matsutake pine mushroom, shamrock, et al		*Anopheles and* *Aedes*◆; *Culex* *quinquefasciatus*●		(Kline et al. 2007, xu et al. 2015)
(*Z*)-jasmone	*Mulberry leaves*		*H.armigera*◆		(Di et al. 2017)
(*Z*)-3-hexenyl acetate, methyl salicylate		*Triticum* *aestivum, et* *al*		*Episyrphus* *balteatus, Harmonia* *axyridis, Hippodamia* *variegate, Coccinella* *septempunctata, Stethorus* *punctum picipes*◆	(Yu et al. 2008; Xie et al. 2014; Maeda et al. 2015; GENCER et al. 2017)
6-Methyl-5-hepten-2-one, *β*-elemene		*Amaranthus* *retroflexus*		*Microplitis mediator*◆	(Yu et al. 2018)
*α*‐pinene and *α*‐copaene		*Zea mays*	FAW	*Chelonus insularis*◆	(Ortiz-Carreon et al. 2019)
*β*-caryophyllene		*Solanum* *tuberosum*	*Bemisia tabaci*◆		(Zhang et al. 2019a)
2-Ethyl-1 hexanol, nonanal		*Triadica* *sebifera*	*Bikasha collaris*◆		(Sun et al. 2019)
Hexanal, methyl propyl disulfide, 1-octen- 3-ol	*Allium and* *Pleurotus*		3rd instar larva and female adult of *Bradysia* *odoriphaga*●		(Zhang et al. 2019b)
(1R)-( +)-*α*-pinene, *α*-caryophyllene, *β*- caryophyllene	Rutaceae		*Diaphorina citri*◆		(Grafton-Cardwell et al. 2013; Wang et al. 2019)
(*Z*, *E*)-*α*-farnesene (49%)、(*E*)-*β*-farnesene (26%), (*Z*,*E*)-*α*-farnesene (7%)		*Nicotiana* *tabacum*	*Helicoverpa* *assulta*●		(Wu et al. 2019)
D-limonene, *β*-ocimene		*Citrus*		*Aphytis melinus*◆	(Mohammed et al. 2019)
Indole		*Oryza sativa*	FAW●		(Ye et al. 2019)
		*Zea mays*	*S.littoralis*●		(Veyrat et al. 2016)
TMTT		*Solanum* *melongena*		*Macrolophus pygmaeus*◆	(Maselou et al. 2019)
(*E*)-Nerolidol	Tea plant		*Empoasca onuki*●		(Chen et al. 2020)
*β*‐caryophyllene	Potato		*Phthorimaea operculella*●	*Trichogramma chilonis*◆	(Munawar et al. 2022)
(*E*)-*β*-farnesene				*Eupeodes corollae*●	(Wang et al. 2022a)
(*E*)-2-hexen-1-ol, 1-octen-3-ol and (*E*,*E*)-2,4-hexadienal	kidney bean		*F. occidentalis*●	*Orius similis* ◆	(Huang et al. 2022)
(Z)‑3‑hexenyl‑acetate	*Z. mays*		FAW◆		(Wang et al. 2023)
*S*‐linalool and *β*‐ocimene		tea plant		*Parapanteles hyposidrae*◆	(Liu et al. 2024)
*α*-humulene	Tomato		*S. litura*●		(Li et al. 2024)
Benzyl Nitrile		tea plant	*Eocanthecona furcellata ***●**		(Qian et al. 2024)

*CPV* Constitutive plant volatiles,  *HIPV* Herbivore-induced plant volatile,  Attraction effect ◆; Repellent effect ●

Plant mineral nutrient traits can reshape the interactions among plants, herbivore insects, and their predators. Li et al. (2024) reported that fluctuations in N levels significantly affect gene expression linked to α‐humulene synthesis, thereby influencing the release of α‐humulene. Tomato plants with sufficient N content exhibit enhanced attractiveness to *S. litura* larvae, resulting in reduced resistance to herbivore attacks. Conversely, inadequate N levels lead to increased accumulation and emission of the α‐humulene (Li et al. 2024). The production of (*E*)-β-farnesene (EBF), a semiochemical emitted by plants to attract aphid predators, is inversely related to N application in cotton (Chen 2013; Wang et al. 2022a). Research on cucumber plants has shown that the application of Si fertilizer after *Helicoverpa armigera* infection increases the production of herbivore-induced plant volatiles (HIPV) and enhances the attractiveness of cucumber plants to predatory beetles, such as *Dicranolaius bellulus* (Kvedaras et al. 2010). Subsequent studies have indicated a significant positive relationship between Si levels in grapes and predator attraction following attacks by European grapevine moths (Connick 2011). Furthermore, the application of Si has been found to regulate the emission of HIPVs by common bean plants infested with two-spotted spider mites, thereby attracting more *Phytoseiulus persimilis* (Islam et al. 2022). Similar results were found in rice, exogenous Si application augments rice's appeal to natural enemies of *Cnaphalocrocis medinalis*, notably reducing HIPVs post-infestation and enhancing attraction to parasitic wasps such as *Trathala flavoorbitalis* and *Microplitis mediator* (Liu et al. 2017). The application of Si can also intensify predation pressures on insects by prolonging their developmental stage, particularly delaying the penetration of newly hatched larvae through the plant’s epidermis and tissues (Kvedaras and Keeping 2007).

## Functions of mineral nutrition in compensatory plant mechanisms after herbivory stress

Although most plants have a comprehensive defense system against herbivores, they are unable to completely avoid being consumed. Plants therefore display compensatory responses to herbivore feeding, such as the promotion of growth and reproduction after herbivory. The ability to regrow and reproduce after an herbivore attack, which is influenced by numerous internal and external elements, is vital for plant survival and embodies a complementary resistance tactic (Erb and Reymond 2019). Minerals absorbed by plants play a vital role in alleviating the adverse effects of herbivory and stimulating compensatory growth; supplementation with mineral nutrients may enhance the efficiency of nutrient absorption and utilization to meet the growth requirements of damaged areas. An increase in leaf N content has been observed in damaged plants, suggesting a potential role for N in plant resilience (Mundim et al. 2012). Similarly, K-treated plants exhibited lower susceptibility to larval infestation, probably because K effectively aids in plant recovery from damage caused by borer larvae (Sarwar 2012). Silicon also promotes the compensatory growth of wheat when attacked by leaf-feeding and root-feeding herbivores and further enables plants to tolerate herbivores by enhancing plant growth (Johnson et al. 2019).

## Concluding remarks and future perspectives

**Fig. 2:**
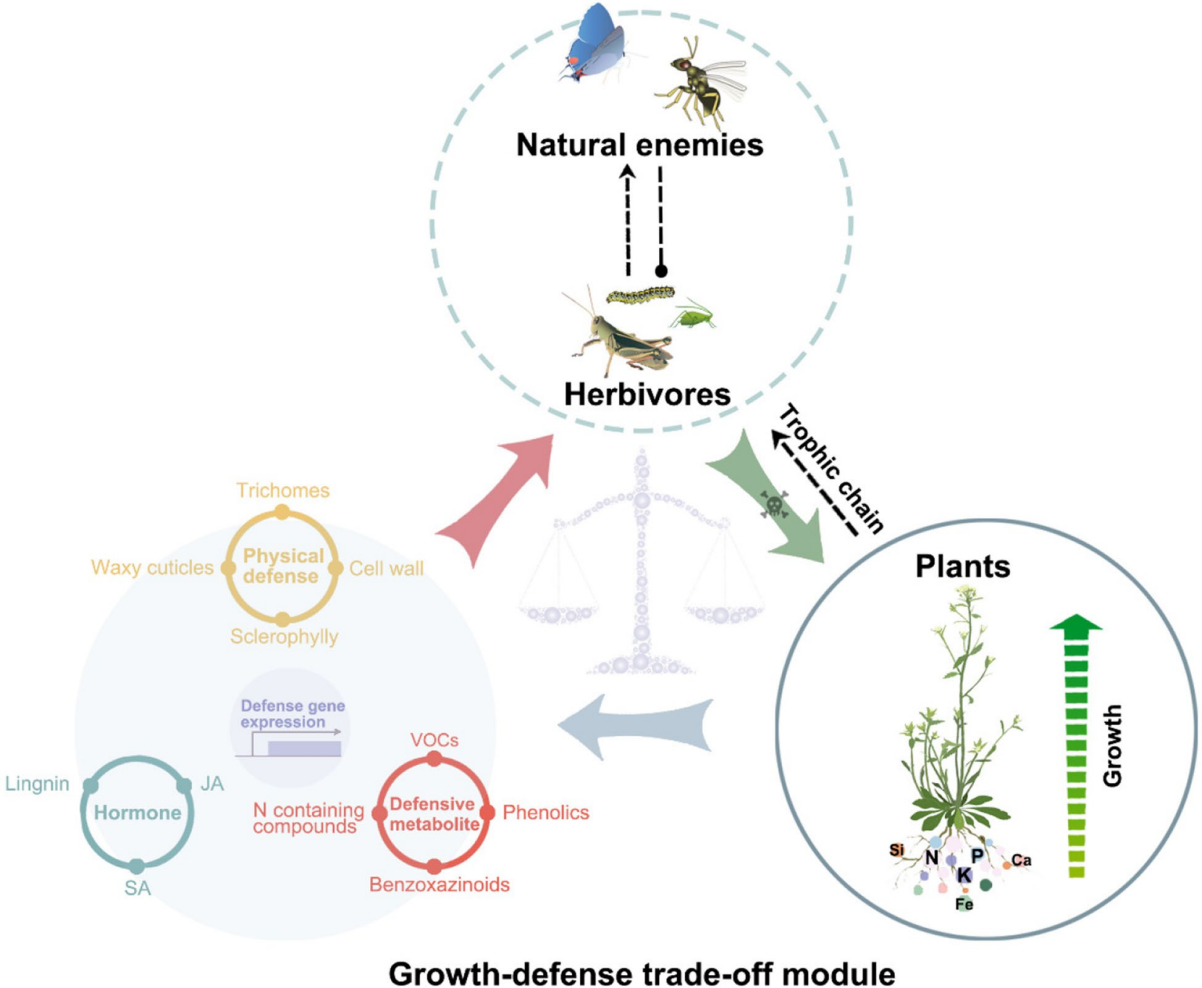
Nutrient-mediated growth-defense trade-off strategies. The three solid circles show the reported functions of mineral nutrients on plant herbivores, including modulation of physical defense, hormonal response, and defensive metabolites. The concentric circles in the middle indicate other defenses mediated by the activation of defense-related genes. The circles of different colors at the plant root represent distinct nutrient elements. VOCs, volatile organic compounds; JA, jasmonic acid; SA, salicylic acid

The simultaneous enhancement of plant growth and herbivore resistance is the holy grail of plant breeding. However, this objective is frequently hindered by the growth-defense trade-off strategies in plants. Over the past few decades, research has significantly advanced our understanding of how nutrient status influences plant resistance to herbivorous insects and the intricate crosstalk between nutrient signaling and insect defense responses (Fig. 2). We currently lack reliable answers to many important questions in this field. One key question that warrants further exploration is how plants integrate multiple environmental signals to prioritize nutrient allocation under herbivore attack and whether these allocation strategies differ according to the type of available nutrients. In addition, the specific mechanisms by which nutrient availability influences the production of defense compounds in plants and how these compounds interact with herbivores to deter feeding will require further investigation. Furthermore, understanding the long-term impacts of plant nutrient status on the evolution of herbivore resistance mechanisms and the ecological consequences of altered nutrient signaling pathways will provide critical insights into the complex interplay between plants and insect pests in natural ecosystems.

Despite a growing body of research exploring how mineral nutrients regulate plant immune responses, there have been conflicting results, particularly in studies involving macronutrients such as N. Such discrepancies may be associated with the comparative costs and advantages of constitutive versus inducible defenses in plants, where metabolic investments are dynamically distributed to optimize survival strategies based on prevailing nutritional conditions. In resource-abundant environments or when nutritional availability is high, plants tend to allocate resources toward growth and development, resulting in relatively low investment in defense mechanisms. Consequently, compared with plants growing in nutrient-poor conditions, nutrient-rich plants are more attractive to insects. However, due to increased nutrient uptake, these plants exhibit higher levels of inducible defense plasticity. In response to herbivore attacks, they are more likely to manifest inducible defenses and compensatory growth, as they possess greater post-attack nutritional resources to synthesize relevant anti-insect metabolites and promote regenerative growth and development.

Bottom-up effects play critical roles in plant-herbivore-natural enemy multiple trophic interactions within ecosystems, and over the past two decades, they have emerged as crucial drivers of integrated pest management (IPM) (Han et al. 2022). Fertilization is an indispensable element in a majority of agricultural practices and is identified as a significant trigger of these bottom-up effects, leading to substantial implications for pest management. The soil nutrient environment can profoundly impact pest populations by altering multitrophic relationships (Han et al. 2022). The nutritional content of crops is a key determinant influencing herbivore performance and fitness, thereby affecting the anti-predator defenses of insect herbivores and subsequently altering their susceptibility to natural enemies like predators and parasitoids. (Islam et al. 2023). Based on the insights gleaned from the above observations, future agronomic practices should focus on refining nutrient management strategies to optimize plant health, resistance, and resilience. Fostering a deeper comprehension of the complex interplay between plant nutrition, herbivore dynamics, and natural enemy interactions will be paramount for enhancing sustainable pest management approaches and promoting balanced agroecosystem health.

## Data Availability

No datasets were generated or analyzed during the current study.
